# Potential of Microbial Diversity of Coastal Sand Dunes: Need for Exploration in Odisha Coast of India

**DOI:** 10.1155/2019/2758501

**Published:** 2019-07-14

**Authors:** Shubhransu Nayak, Satyaranjan Behera, Prasad Kumar Dash

**Affiliations:** Odisha Biodiversity Board, Regional Plant Resource Centre Campus, Ekamra Kanan, Nayapalli, Bhubaneswar 751015, Odisha, India

## Abstract

Coastal sand dunes are hips and strips formed by sand particles which are eroded and ground rock, derived from terrestrial and oceanic sources. This is considered as a specialized ecosystem characterized by conditions which are hostile for life forms like high salt, low moisture, and low organic matter content. However, dunes are also inhabited by diverse groups of flora, fauna, and microorganisms specifically adapted to these situations. Microbial groups like fungi, bacteria, and actinobacteria are quite abundant in the rhizosphere, phyllosphere, and inside plants which are very much essential for the integration of dunes. Microorganisms in this ecosystem have been found to produce a number of bioactive metabolites which are of great importance to agriculture and industries. Many species of arbuscular mycorrhizal fungi and Rhizobia associated with the roots of dune flora are prolific producers of plant growth promoting biochemicals like indole acetic acid. In addition to that bacteria belonging to* Pseudomonas* sp.,* Gammaproteobacteria* have been found to have antagonistic activity towards plant pathogens like* Rhizoctonia solani*,* Pythium ultimum, Fusarium oxysporum,* and* Botrytis cinerea*. Many neutrophilic and alkaliphilic eubacterial species, endophytic fungi from dunes have proved their ability for the production of extracellular enzymes like cellulase, pectinase, amylase, protease, tannase, chitinase, etc., which are of great importance to various industries. In this context, it is relevant to observe that the state of Odisha in India has a 480km long coast having numerous sand dunes. These dunes are rich in floral and faunal diversity. However, a comprehensive study is yet to be taken up to explore the microbial diversity and their bioactive potential in this region. The current review sheds light on the enormous potential of sand dune microorganisms in the coast and surfaced the idea and need for such exploration in the state of Odisha, India.

## 1. Introduction

Coastal sand dunes are described as mounds and narrow strips of sand with distinct boundaries determined by the sea and landward limits of sand transport. Sand dunes are widely distributed across the globe, covering 6×10^6^ km^2^ of its land surface. This specialized ecosystem is considered to be unique and different from inland dunes due to the effect of sea [1–3]. These are natural barriers against the wave action of the sea, protecting the shoreline and natural habitats or developed areas inland [4]. These structures are also the basis of important ecosystems, supporting valuable communities of plants and animals. As such, they provide resources and shelter for aboriginal people, generating cultural values that remain important today. Sand dunes are now recognised as integral parts of our beach systems with intrinsic biodiversity values [5]. The ecosystem of sand dunes is too hostile for the establishment of normal life forms and also does not support dense and very diverse vegetation. However, due to unusual circumstances, this environment harbours atypical floral, faunal, and microbial diversity [6]. Coastal dunes have been reported to contain a larger diversity of agriculturally, industrially, and pharmaceutically important bacteria, fungi, actinomycetes, and other groups of microbes [7–15]. Despite these facts, unlike desert dunes, coastal dunes have generally been neglected and attracted little attention from investigators for study on their biodiversity. Particularly, the potential of microbial diversity has not been exploited much in India. In Odisha, the area has still been remained untouched despite of having a coast line of about 480km. Hence, it has become very important to highlight the bioefficacy of such novel microbial diversity and their potential role in various fields with a context to Odisha. Further, with the mandate of the “Convention on Biological Diversity,” the Biological Diversity Act 2002 implemented by the Government of India u/s 41 also puts provision for the documentation of microbial resources along with flora, fauna, and associated traditional knowledge. It has, therefore, become very much essential to explore microbial potential in unattended ecosystems like the coastal sand dunes in Odisha. The current review sheds light on such issues in a concise way.

## 2. The Extraordinary Ecosystem and Microbial Relevance

The main composition of coastal sand dunes is permeable sand materials which act as biocatalytic filters for various types of materials advected by currents and winds, including dissolved and particulate organic matter derived from living and dead biomass of terrestrial or marine origin [16]. These are dynamic and disturbance prone habitats which undergo continuous alteration in topography by sand movement as well as physicochemical features mainly due to strong wind and wave action. These are generally characterized by infertile soil along with saline mist, salt spray, dryness, lack of water holding capacity, desiccation, high light intensity, nutrient deficiency, sand erosion, sand accretion, and other transitional factors [17]. Such environmental stresses not only affect the pattern of vegetation and faunal diversity, but also the dynamics and composition of microbial community.

Diversity of microorganisms in the dune ecosystem may be viewed from two angles, bulk sand and plant associated. In sand dune ecosystems, lack of abundant vegetation cover constrains the soil carbon availability [18] and, hence, affects the microbial biomass [19]. Subtidal sands harbor rich microbial diversity due to the accumulation of both organic and inorganic materials. In contrary to this, supratidal sands which include sand dunes have low organic matter content and concentrations of other reactive substances required to support biological processes, therefore, having less diversity of microbial communities. Microbial biomasses like carbon and nitrogen are higher in the humic layer than the underlying soil layer in sand dune forest soil [20]. However, Probandt et al. [21] estimated that a single sand grain may contain 10^4^ to 10^5^ microbial cells. Predominant population of* Acidobacteria* and* Proteobacteria* have been isolated from humic soils of upland and lowland* Casuarina* and lowland* Hibiscus* successional forests in a coastal sand dune ecosystem of Taiwan, where* Bradyrhizobium* related clones were abundant in* Casuarina* forest soils [22]. The composition of the dune soil microbial community also varies with abiotic stresses, especially soil salinity [19]. The overall interaction in the habitat may impact hydrological, sedimentological, and biogeochemical processes that occur throughout the whole beach system. Though the dynamic environmental parameters in coastal sands pose a challenge to many microbial communities, still a rich bacterial community is supported by the high diversity of niches where few but tolerant and well-adapted resident types are naturally selected [23]. Gobet et al. [24] found only 3 to 5% of all bacterial types were present at all the seasons in a given depth zone of sand soil, but 50-80% of them belonged to the most abundant types. Sridharan et al. [25] isolated 24 fungal species belonging to* Zygomycetes*,* Ascomycetes,* and* Deuteromycetes* from the dune soils of Tamil Nadu coast.

## 3. Microorganisms Associated with Root and Rhizosphere

Plantations present on coastal sand dunes vary in structure and composition and these variations may alter soil microbial communities [26]. Rhizosphere is the thin soil layer immediately surrounding plant roots and microorganisms which are coexisting in the rhizosphere are known as rhizospheric microorganisms. Plants in dunes and in general have specific rhizospheric microflora which depends on the influx of mineral nutrients to roots and the abundance, density, composition, and diversity of plant-derived exudates. Interactions with microbes like Arbuscular mycorrhizal fungi in the roots appear crucial in obtaining inorganic nutrients or growth-influencing substances. Plant-associated bacteria increase the ability of plants to utilize nutrients from the soil by increasing root development, nitrate uptake, or solubilizing phosphorus and to control soilborne pathogens. Successful restoration of plants like* Ammophila arenaria* in nutrient-poor saline dunes even depended on plant-microbe symbiosis like nitrogen-fixing bacteria [27].

Some perennial shrubs like* Artemisia monosperma *and* Retama raetam* have created favorable niches for microorganisms where the rhizospheric microbial mass was found to be 234.6 and 173.1 *μ*gCg^−1^ dry soil [17]. Rhizospheric soil of* Calystegia soldanella* (beach morning glory) and* Elymus mollis* (wild rye) found in South Korean dunes have been found to be enriched with 27 different established genera of bacteria [27]. A total of 47 rhizobacterial strains (including 22 fluorescent* Pseudomonads*) could be isolated from 5 rhizosphere soil samples collected from the coastal sand dunes in Uthandi Beach, Chennai [7]. Abundant population of siderophores producing bacteria belonging to* Bacillus *spp.,* Streptomyces *spp.,* Pseudomonas *spp.,* Renibacterium *spp.,* Corynebacterium*,* Azotobacter *spp.,* Kurthia *spp.,* Brochothrix *spp., etc. could be isolated from rhizosphere of sand dune creeper* Ipomoea pescaprae* found in Miramar, Goa, located on the west coast of India [2]. Extreme halo-tolerant novel bacterial species named as* Halomonas malpeensis* sp. nov. has been recently isolated from the rhizosphere sand of a coastal sand dune plant which could grow in the presence of 0.5 to 5* *% (w/v) NaCl (growth up to 20* *% NaCl) and had a wide range of growth conditions [28].

## 4. Prevalence of Arbuscular Mycorrhizal Fungi (AMF)

Establishment of vegetation in stressed environments like sand dunes have been promoted by Arbuscular mycorrhizal fungi (AMF), a special group of fungi which can enhance uptake of plant nutrient and water and protect plants from root herbivores and pathogens. Particularly, this microbial group plays an important role in stabilization and rehabilitation of dunes by a way of producing glomalin and development of a vast network of hyphae which ultimately prevent soil erosion by the formation of wind-resistant soil aggregates. The AMF belong to the phylum Glomeromycota, which includes about 270 described species. These fungi are an important link in the soil-plant interface because they form a mutualistic symbiosis with plant roots of over 80% of vascular plants and act as efficient facilitators for the absorption of nutrients by their host plants, including most inaccessible nutrients (e.g., phosphorous), increase in the complementarities of nutritional resources, ensure greater tolerance to biotic and abiotic stresses, and contribute to the stabilization of soils due to the formation of stable soil aggregates [29].

Unvegetated sites in dunes generally lack propagules of AMF, but when culms of plants (carrying propagules of AMF) like* A*.* breviligulata* transplanted in such area, the roots became mycorrhizal in less than a year after planting. The province lands area of Cape Cod National Seashore, Massachusetts, is such a site where early and later sporulating species of AMF were isolated in the dune sites which suggested an apparent succession of AMF [30]. In another experiment in dunes of Mexico, AMF colonization in transplanted seedlings of* Chamaecrista chamaecristoides* was found to be higher in foredunes than mobile dunes. Development of hyphae, arbuscules, and vesicles were observed in 100, 46, and 20% of such plants, respectively [31]. Several* Glomus*,* Scutellospora,* and* Acaulospora* species have been found to be associated with* A*.* arenaria* ssp.* arenaria* in European coastal dunes [32]. Particularly, maritime dune soils of Denmark were found to contain G*. irregulare* (present in 73.6% of samples),* G. aggregatum *(9.0%), and* G. eburneum *(7.1%) which had less sporulating ability in the field [33]. Brazilian dunes have specifically exhibited hot spot for mycorrhizal diversity where species richness of AMF was observed more in maritime dunes than the fluvial dunes. Canonical correspondence analysis (CCA) conducted for AMF population in maritime dunes of Bahia showed that the content of coarse sand was correlated with the occurrence of* Acaulospora scrobiculata* (Acascrob),* Gigaspora* sp.1 (Gigsp1),* Glomus brohultii* (Globro),* Glomus macrocarpum* (Glomac),* Glomus microcarpum* (Glomic),* Glomus* sp.2 (Glosp2),* Glomus trufemii* (Glotruf), and Intraornato- spora intraornata (Intint). On the other hand, the content of K, P, Ca, Fe, pH, Na, clay, and fine sand influenced the occurrence of the following AMF species:* Acaulospora mellea* (Acamel),* Acaulospora foveata* (Acafov),* Ambispora appendicula* (Ambapp),* Claroideoglomus etunicatum* (Claetu),* Funneliformis halonatus* (Funhal),* Fuscutata heterogama* (Fushet),* Fuscutata rubra* (Fusrub),* Gigaspora gigantea* (Giggig),* Gigaspora margarita* (Gigmar),* Gigaspora rosea* (Gigros), and* Racocetra fulgida* (De Assis et al., 2016). Similarly, Itapiruba (southern), Joaquina (intermediate), and Praia Grande (northern) parts of Brazilian coast exhibited diversity of AMF belonging to 25 species were recovered belonging to seven genera and five families in the Glomeromycota. However, the diversity did not vary among sites despite being geographically distant (150 km) from each other [34]. Revegetated mined dunes in north east coast (Mataraca, Paraíba State) of Brazil were even found to be rich in 34 species of AMF, of which 29 were identified in field samples and five after trap culturing, with the greatest diversity and richness found in the dune revegetated 8 years ago.* Gigaspora margarita* was the most dominant species in those sites [35]. In one of the hot spot areas in this coastal part of Brazil, higher AMF spore density was observed in the arboreal dune compared to the shrubby and herbaceous dunes, while the highest number of infective propagules was observed in the herbaceous dune, followed by the shrubby and arboreal dunes. However, AMF species richness and AMF root colonization were similar in all dunes. In this site,* Funneliformis halonatus* was the indicator species of the arboreal dune;* Ambispora appendicula*,* Gigaspora gigantea,* and* Paradentiscutata maritima* were the indicator species of the shrubby dune, while two undescribed* Cetraspora* species and* Sclerocystis sinuosa* were the indicator species of the herbaceous dune [36].

Species like* G*.* mosseae*,* G*.* dimorphicum*,* G. fasciculatum*,* Gigaspora gigantea*, and* Acaulospora taiwania* have been associated with* Ipomoea pes-caprae* (L.), a common sand creeper in severely disturbed west coast sand dunes of India [37, 38]. Another economically important plant* Drimia indica* (Roxb) Jessop. (Family: Hyacinthaceae), also known as Indian squill, found in the same region has been found to be colonized with 9 species of AMF [39]. A great diversity of AMF consisting of 36 species belonging to genera* viz. Acaulospora, Diversispora, Entrophospora, Gigaspora, Glomus, Rhizophagus, Sclerocystis, *and* Septaglomus *were isolated from the rhizosphere soil of* Distichlis distichophylla, Cucumis humifructus, Tephrosia purpurea, *and* Prosopis juliflora *growing in Manapaddu coastal region of Tamil Nadu [40].

## 5. Sparse Microfungal Diversity

Abundance of microfungi in the soil of coastal sand dunes is restricted due to low amount of carbon where few studies have enumerated their biodiversity. However, a great number of saprophytes mainly the decomposers have been isolated from the top soil where the amount of organic matter remains high. Erstwhile, studies indicated the presence of common cellulose decomposing fungi like* Cephalosporium acremonium*,* Fusarium* spp.,* Penicillium* spp. and* Trichoderma viride*,* Chaetomium*,* Doratomyces*,* Humicola, Gliomastix,* and Sordaria. Uncommon species like* Acrospeira levis*, conidial* Apiospora montagnei*,* Trichocladium asperum,* and* Volutella ciliata* have also been obtained in the same study [41]. Some mobile (yellow) dunes of Europe possessed specialist fungi, e.g.,* Peziza ammophila*,* Melanoleuca cinereifolia*, the brown-rot fungus* Pilidium concavum, *and the white-rot fungus* Phanerochaete *spp., and many other saprophytic fungi belonging to Basidiomycota like* Melanoleuca cinereifolia, Phlebiella christiansenii*,* Phanerochaete*,* Melanoleuca cinereifolia, Phlebiella christiansenii*,* Phanerochaete *spp. etc. have also been isolated from sand dunes [42]. More than 100 species of fungi including sugar fungi have been enumerated from the coastal dunes of Odisha, the majorities of which belonged to the genus* Aspergillus* followed by* Penicillium *and* Trichoderma* [43, 44].* Penicillium* population was evenly distributed in those dunes having more than 40 species [45].

## 6. Macrofungi in Coastal Dunes of India

Macrofungi constitute important natural resource owing to their major role in decomposition, nutrient cycling, mutualistic association, and other advantages (nutrition, medicine, and metabolites). In spite of saline conditions and disturbances, coastal sand dunes may consist of 29% of macrofungi. Sand dunes of Karnataka coast are especially rich in the diversity of macrofungi harbouring some eminent species like* Coprinus plicatilis*,* Crepidotus uber*,* Dacryopinax spathularia*,* Ganoderma lucidum*,* Hexagonia tenuis*,* Lentinus squarrosulus*,* Lycoperdon utriforme*,* Marasmius kisangensis*,* Microporus xanthopus* and* Thelephora palmate, Amanita *spp.*, Collybia dryophila *var.* extuberans*, etc. [46, 47]. Spatial and temporal analysis carried out by Ghate and Sridhar [48] yielded 64 species of macrofungi belonging to 34 genera in five coastal sand dunes (CSDs) of southwest India, where* Marasmius *was the major genus (7 spp.) followed by* Agaricus *(5 spp.),* Lentinus *(4 spp.), and* Lepiota *(4 spp.).

## 7. Diversity of the Indispensable Endophytic Microorganisms

This group of microorganisms asymptomatically lives inside the plant body and generally impart beneficial effects to the plant such as growth promotion, protection from biotic and abiotic stress, etc. [49, 50]. Tropical endophytic fungal inventory is turning to be an important component of fungal diversity estimation. Other than AMFs, the coastal sand dune vegetation might depend on endophytic fungi assemblage to cope with extreme conditions. Both bacterial and fungal endophytes have been isolated from roots, vegetative parts, and propagules of plants thriving in coastal sand dunes. Studies conducted in the Korean dunes have enumerated a great diversity of bacterial endophytes, where as many as 21 different bacterial genera could be isolated alone from the roots of two plants* viz. soldanella* (beach morning glory) and* Elymus mollis* (wild rye) [27]. Bacterial species belonging to* Pseudomonas*,* Chryseobacterium*,* Microbacterium*,* Paenibacillus*,* Acinetobacter,* and* Lysobacter* were found to form the majority of the root endophytic bacteria. The dune flora also inhabited by diverse fungal groups where* Penicillium* and* Fusarium* were found to be dominant species [10]. Fungal strains belonging to thirteen orders* viz.* Capnodiales, Eurotiales, Glomerellales, Helotiales, Hypocreales, Mortierellales, Onygenales, Ophiostomatales, Pleosporales, Polyporales, Russulales, Saccharomycetales, and Xylariales could be isolated from coastal sand dune plants like* Argusia sibirica*,* Calystegia soldanella*,* Elymus mollis*,* Lithospermum zollingeri*,* Raphanus sativus*,* Salsola collina*,* Zoysia macrostachya,* and* Zoysia sinica* [51].

Plants in the southern and west coast sand dunes of India are found to be abundantly associated with endophytic microorganisms. The common sand binder* Ipomoea* spp. in this region was found to be mutually associated with 46 morphologically different bacterial strains some of them having Plant Growth Promoting (PGPR) property [8].* Ipomoea pes-caprae *along with* Launaea sarmentosa *and* Polycarpaea corymbosa *were also associated with dominant root endophytic fungi like* Chaetomium globosum *and* Torula caligans*. In a pilot trial by Beena et al. [37, 38],* Acremonium was *found to be the dominant endophyte having no negative impact on the arbuscular mycorrhizal fungal colonisation and reproduction. Forty-six fungal taxa comprising 6 ascomycetes, 33 mitosporic fungi, 2 zygomycetes, and 5 sterile morpho species could be recovered from 3 age classes (seeds, seedlings, and mature plants) and 5 tissue classes (cotyledons, seed coats, roots, stems, and leaves) of coastal sand dune legumes* Canavalia cathartica *and* Canavalia maritima*. The most dominant endophyte,* Chaetomium globosum*, colonized over 50% of the root, stem, and leaf segments of* C. maritima *and over 50% of the root segments of* C. cathartica* [52].

## 8. A Brief Pause on the Role of Microbes for the Integration of Dune Ecosystem

In the foregoing sections, it has been established by fact that despite being a stringent environment, coastal sand dunes have a rich microbial diversity as the integral part of that ecosystem. As mentioned in these discussions, the various groups of fungi and bacteria are responsible for the successful succession and establishment of vegetation and subsequently fauna in the dunes. Microorganisms are fundamental components of any terrestrial ecosystem due to their key role in organic matter decomposition, nutrient cycling, and the development of soil structure, especially in sand dune environments [3]. The increase in number and diversity of microorganisms is supposed to be responsible for the formation of stable aggregates in unstable to stable dunes. One of the multiple reasons for this may be due to the secretion of extracellular complex polysaccharides by certain group of bacteria in response to mycorrhizal activity and another may be the formation of mucilage by* Nostoc* spp. which binds the sand particles to help other microorganisms [6]. The AM fungi themselves are known to improve the soil structure as well as stability due to the formation of sand aggregates [53]. They are responsible for three distinct phases of aggregates: (a) hyphae entangle the soil particles; (b) roots and hyphae create conditions congenial to form microaggregates in soil; and (c) roots and hyphae enmesh and bind the microaggregates to larger macroaggregates [54]. Polysaccharide secretions of AM fungi firmly adhere to the microaggregates [55]. Other than stabilizing the dune structure, microorganisms like* Bacillus* and* Pseudomonas* spp. produce low-molecular-weight, high-affinity iron chelators termed siderophores which reduce the formation of Fe^+3^ ions which in turn reduces the chances of iron deficiency in the rhizosphere [2].

## 9. Beneficial Biopotential of Sand Dune Microbes

The environmental conditions prevailing in the sand dune ecosystems make the microorganisms and other living forms more competent and may have good potential of bioactivity. So bioprospecting of coastal sand dune microorganisms is a worthy one [7]. The proven ability to produce numerous secondary metabolites by various groups of microorganisms inhabiting coastal sand dunes has already shown the path to be exploited for use in agriculture, industries, and pharmaceuticals.

### 9.1. Agricultural Importance

Diverse group of Plant Growth Promoting Rhizobacteria (PGPR) has been isolated from sand dunes in Chennai coast which were found to produce extracellular plant growth hormone indole acetic acid (IAA). These bacteria have demonstrated for their remarkable ability to increase seedling length and seedling weight of wheat, green gram, mustard, black gram, and kidney bean through seedling growth chamber studies [7, 8]. Rhizobia isolated from wild legumes growing in sand dunes of West coast India not only increased nodulation and nitrogen fixation, but also induced higher root and shoot mass when inoculated in cowpea (*Vigna unguiculata*), green gram (*Vigna radiata*), black gram (*Vigna mungo*), and horse gram (*Macrotyloma uniflorum*) [9]. Gibberellins also produced by* Gibberella fujikuroi* from 9 sand dunes of Korea could increase root shot length of* Waito-c* rice [10, 11]. Shin et al. [14] found* Gammaproteobacteria* constituting 65.9% and members of* Pseudomonas* constituted 49.5% of the total isolates which were endophytic to sand dune plants like* Lathyrus japonica*,* Vitex rotundifolia*,* Carex kobomugi*,* Artemisia fukudo*,* Messerschmidia sibirica*,* Glehnia littoralis*, etc. Among them, seven strains were shown to produce indole acetic acid (IAA), 33 to produce siderophores, 23 to produce protease, 37 to produce pectinase, and 38 to produce chitinase. Growth promoting fungi may constitute more than 85% of total fungal population in particular sand dunes. Enhancement of root and shoot length, root and shoot weight of maize plant could be achieved by treatment of extract of endophytic* Phoma* species isolated from* Tinospora cordifolia* and* Calotropis procera* [12]. Similarly, growth of* Waito-c* rice has been enhanced by* Penicillium citrinum *isolated from* Ixeris repens* which extracellularly produced active gibberellins, GA1, GA3, GA4, and GA7 (1.95 ng/ml, 3.83 ng/ml, 6.03 ng/ml, and 2.35 ng/ml, respectively).

Other than the production of growth enhancing phytohormones, microorganisms associated with roots can protect plant growth through the biological control of plant pathogens or they can do both. There are 20 different biocontrol PGPR strains commercially available in the market at present. Many strains of* Pseudomonas* spp. and* Gammaproteobacteria* having such broad spectrum activity have been isolated by Shin et al. [14], among which 25 strains were antagonistic towards* Rhizoctonia solani*, 57 strains were antagonistic towards* Pythium ultimum*, 53 strains were antagonistic towards* Fusarium oxysporum*, and 41 strains were antagonistic towards* Botrytis cinerea*.* Pseudomonas *spp. AMET 6007 specifically exhibited 32% control over Sheath blight pathogen (*Rhizoctonia solani*) of rice by their antagonistic mechanisms such as competition for iron (production of siderophores), production of volatile (hydrogen cyanide, HCN) and diffusible metabolites in addition to their ability to produce IAA [56]. Similar dual property has been exhibited by rhizobacteria obtained from plants growing in the coastal sand dunes located along East Coast of Korea. Out of 1330 rhizobacteria screened from 11 different plants from this area, 23 strains were able to produce auxin and had spectrum of antagonism toward various phytopathogenic microbes [57]. Not only that, obligate endospore-forming parasitic bacterial strains like* Pasteuria penetrans* isolated from European fore dune flora* Ammophila arenaria* showed the potential to control aggressive nematode like* Meloidogyne *spp. [4]. Sangeetha [13] isolated 70 actinobacterial strains, most of them which exhibited PGP activity and inhibited the mycelia growth of* Rhizoctonia solani*,* Fusarium oxysporum,* and* Sclerotium rolfsii*. Strain AMET053 which was identified as* Streptomyces rochei* produced highest zone of inhibition of 9mm and protected tomato plant against* Fusarium* wilt disease in green house.

### 9.2. Production of Industrial Enzymes

Microorganisms mainly bacteria, fungi, and actinomycetes are active producers of many extracellular enzymes like cellulase, pectinase, amylase, protease, tannase, chitinase, etc. These enzymes are gold mines to be used in various industries for the conversion of biological waste residues like agrowaste to useful products. A large number of such microorganisms have been isolated from marine and coastal environment. There are also few reports of collection of such microbial strains from coastal sand dunes which could produce these enzymes in vitro and showed the potential for industrial use. Many neutrophilic and alkaliphilic eubacterial species from rhizosphere* Ipomoea pes*-*caprae* and* Spinifex littoreus* are having special capabilities of producing multiple hydrolytic enzymes as mentioned above. The majority of such neutrophilic bacteria belonged to* Bacillus* sp., while alkaliphilic bacteria were gram-positive irregular rods belonging to* Brevibacterium*,* Brochothrix*,* Cellulomonas,* and* Microbacterium*. These bacterial populations are higher during premonsoon period due to the process of degradation of shredded foliages [58]. Many endophytic bacteria isolated from Tae-An area (Baramarae, Sambong, Shindu, and Hagam), Chungnam Province of Korea produced important industrial enzymes like protease, pectinase, and chitinase. These bacteria belonged to* Pseudomonas* spp.,* Rahnella* spp.,* Rhizobium* spp.,* Xanthomonas* spp.,* Pantoea* sp.,* Erwinia* spp.,* Chryseobacterium* spp.,* Bacillus* spp., and* Acinetobacter* spp. [14].* Penicillium* spp. strain IS-07 isolated from dunes of Brazilian coast could produce cellulase enzymes (Carboxymethyl-cellulase 2274 U/L and Filter-paperase 128 U/L) by using sugarcane wastes in a fermentation process [15]. Another Brazilian strain* Trichoderma* spp. IS-05 from the dune soil of Guaibim beach produced 564.0 UL^−1^ of cellulase, obtained after 2 days by fermentation of wheat bran [59]. Other than fungi and bacteria,* Actinobacteria* from the sand dune rhizospheric soil of Chennai coast of India were screened by Sangeetha [13] for the production of lytic enzymes. Out of 70 morphologically distinct strains, 34 strains produced amylase, 11 strains produced tannase, 30 strains produced chitinase, 61 produced gelatinase, 51 produced caseinase, and 24 produced pectinase. The extraordinary capability of sand dune microbes for the production of such important enzymes can be harnessed in specific industries and commercial scale production of such enzymes may be carried out through fermentation process.

## 10. Coast, Coastal Dunes and Its Microflora in Odisha

The state of Odisha is situated in the eastern part of India and is one of the four maritime states bordering the Bay of Bengal. The state has a coastline of about 480km constituting 8% of the Indian coastline which stretches from the Udaipur village (21° 31' N; 87° 34' E) bordering West Bengal in the north to the marshes of Ichapuram and Bahuda estuary bordering Andhra Pradesh in the south (19° 02' N; 84° 47' E). This includes the luxuriant Bhitarkanika mangrove vegetation, Mahanadi delta, and the coastal Chilika Lake. There are seven coastal districts, namely, Ganjam, Puri, Khordha, Jagatsinghpur, Kendrapara, Bhadrak, and Balasore, consisting of approximately 250 coastal fishing villages and about 25 fish landing centres along the coast of Odisha. This coast is bestowed with a variety of habitats, such as sand dunes, tidal creeks, backwaters, brackish water lagoons, estuaries, mangroves, mudflats, and salt marshes. The Exclusive Economic Zone (EEZ) of the state has been estimated at 172,000km^2^ ([60]; http://iomenvis.nic.in).

Although there has been no specific investigations carried out to study the biodiversity of the sand dunes of Odisha coast, few reports have certainly indicated the existence of rich diversity of flora, fauna, and microbial resources in this specialized ecosystem. The floristic composition in the coastal line was dominated by some of the larger plant families including Asteraceae, Fabaceae, Poaceae, and Convolvulaceae [61]. Particularly in the dunes, at least 55 species of medicinal plants having ethnobotanical use have been identified by Pattanaik et al. [62]. This much is enough to indicate the existence of rich microbial diversity associated either with the rhizosphere or present as endophytic mode. As far as microbial diversity is concerned, novel fungal and bacterial strains have been isolated from various coastal habitats of Odisha like Bhitarkanika mangrove area [63]. Even arbuscular mycorrhizal (AM) fungi belonging to genera* Glomus*,* Acaulospora*,* Gigaspora*,* Scutellospora,* and* Entrophospora *were recorded from different salinity zones of Bhitarkanika mangrove ecosystem [64]. However, Panda et al. [43] have conducted a study particularly in the dunes of southern coast of Odisha in* Anacardium* and* Casuarina* plantation area. Fungal community in sand dunes with* Anacardium *plantation was found to be of 45 genera & 114 species on surface and 41 genera & 93 species on subsurface. Sand dune with* Casuarina *plantation was having 36 genera & 78 species of fungi on surface and 38 genera & 85 species on subsurface. Barren sand dune possessed 54 genera & 91 species on surface and 46 genera and 80 species on subsurface. Considering a vast coast line and diversity of dunes in Odisha, a large gap can be clearly observed regarding the exploration of microbial diversity particularly in sand dunes. Specific efforts need to be initiated to explore numerous possibilities in this unattended ecosystem.

## 11. Conclusion

Current discussions have brought out the enormous potential of coastal sand dunes in terms of having useful microbial diversity. Such hostile habitats around the world have been explored and numerous useful microorganisms have been isolated and identified which possessed the ability to produce many important biochemicals. Particularly, the Arbuscular Mycorrhizal Fungal (AMF) group has been predominant in association with plants which could be exploited and applied in the agricultural field. The state of Odisha in spite of having such a long and biodiversity rich coast still needs to be explored for such novel microorganisms. More than 60% population of Odisha is dependent on agriculture. Most of the farmers use chemical fertilizers and pesticides during agricultural operations which are of higher cost and sometimes those are beyond the buying capability of farmers. In this regard, the discovery of novel microorganisms (fungi, bacteria, and actinobacteria) from this ecosystem in Odisha having plant growth promotion and biological control properties could do wonders towards the idea of sustainable agriculture in the whole state. The extraction of an array of industrial enzymes might lead to the establishment of relevant industries like bioethanol, detergent plats, etc. which may generate jobs in addition to use of agrowaste and other byproducts.

Furthermore, useful and efficient microorganisms could be protected from biopiracy and financial benefits could be generated through patent rights by constitution of Biodiversity Management Committees (BMCs) as per provisions mentioned U/S 41 of the Biological Diversity Act 2002 of Government of India. Section 41(1) of the Act says, “Every local body shall constitute a Biodiversity Management Committee within its area for the purpose of promoting conservation, sustainable use and documentation of biological diversity including preservation of habitats, conservation of land races, folk varieties and cultivars, domesticated stocks and breeds of animals and microorganisms and chronicling of knowledge relating to biological diversity.” In accordance with “Nagoya Protocol,” to which India is also a party, Government of India has notified “Guidelines on Access to Biological Resources and Associated Knowledge and Benefits Sharing Regulations, 2014.” Along the strength of these guidelines, the BMCs have to get the benefit sharing from the utilisation of any such microbial resources from the coastal sand dunes. As microorganisms cannot be viewed with naked eye, scientific investigations need to be carried out to explore the microbial diversity and preserve useful ones for their utility for specific purpose. Hence, a comprehensive effort is required for the enumeration of microbial diversity in coastal sand dunes of Odisha and the bioactive potential need to be exploited for various applications. The Forest Department of Odisha State has recently initiated efforts for such study through Odisha Biodiversity Board, Bhubaneswar. A project has been sanctioned for a comprehensive study on the flora, fauna, and microorganisms in the coastal sand dunes of Odisha. The outcome of the project may be utilized in the management plan of coasts by the State Government of Odisha.
